# Role of RUNX3 in Suppressing Metastasis and Angiogenesis of Human Prostate Cancer

**DOI:** 10.1371/journal.pone.0086917

**Published:** 2014-01-24

**Authors:** Feifei Chen, Meng Wang, Jin Bai, Qinghua Liu, Yaguang Xi, Wang Li, Junnian Zheng

**Affiliations:** 1 Jiangsu Key Laboratory of Biological Cancer Therapy, Xuzhou Medical College, Xuzhou, Jiangsu, China; 2 School of Pathology, Xuzhou Medical College, Xuzhou, Jiangsu, China; 3 Mitchell Cancer Institute, University of South Alabama, Mobile, Alabama, United States of America; 4 The Affiliated Hospital of Xuzhou Medical College, Xuzhou, Jiangsu, China; Northwestern University, United States of America

## Abstract

RUNX3 (runt-related transcription factor-3) has been reported to suppress tumor tumorigenesis and metastasis in different human cancers. In this study, we used tissue microarray (TMA) to determine the significance of RUNX3 in prostate cancer progession. Our results showed ectopic expression of RUNX3 in prostate cancer tissues when compared with tumor adjacent normal prostate tissues, and reduced RUNX3 staining was significantly correlated with TNM stage. Moreover, we demonstrated that RUNX3 overexpression inhibited prostate cancer cell migration and invasion resulting from the elevated upregulation of tissue inhibitor of matrix metalloproteinase-2 (TIMP-2), which subsequently inhibited metalloproteinase-2 (MMP-2) expression and activity in vitro. Knock down of RUNX3 expression broke up the balance of TIMP-2/MMP-2, whereas silence of TIMP-2 resulted in the inhibition of MMP-2 expression in prostate cells. We also showed that restoration of RUNX3 decreased vascular endothelial growth factor (VEGF) secretion and suppressed endothelial cell growth and tube formation. Strikingly, RUNX3 was demonstrated to inhibit tumor metastasis and angiogenesis in vivo. Altogether, our results support the tumor suppressive role of RUNX3 in human prostate cancer, and provide insights into development of targeted therapy for this disease.

## Introduction

Prostate cancer is the second leading cause of cancer death among men in the USA [1]. Surgical intervention is still the most effective treatment for primary prostate cancer, although about 30% of prostate patients occur disease relapse and/or metastasis after initial therapies, resulting in the relatively short survival period (12–15 months) [2]. Metastasis is an extraordinarily complex process, including cancer cells migrate out of primary tumors and invade into neighboring tissue, intravasate into the blood or the lymphatic circulation, survive in the blood stream, and target specific organs to initiate metastatic outgrowth [3]. It is of importance to better understand the mechanistic basis of tumor metastasis by identifying the key molecules involved in this process, which will provide insights into development of more efficacious strategies to prevent tumor progression.

Runt domain family, consisting of RUNX1, RUNX2, and RUNX3, are master regulators of gene expression in cell proliferation and differentiation. RUNX family proteins contain the well conserved domain with 128 amino-acids region (Runt domain) and form a stable complex with PEBP2b/CBFb to exert its transactivation ability [4]. All three RUNX family members play important roles in normal developmental processes and tumotigenesis [5]. RUNX proteins regulate the expression of cellular genes by binding to promoters or enhancers of target genes related to cell-fate decisions, which become deranged in cancer cells [6].

Among the three RUNX family members, RUNX3 was originally cloned as AML2 and is localized on chromosome 1p36.1. RUNX3 was first reported to correlate with the genesis and progression of human gastric cancer as a tumor suppressor [7]. Besides gastric cancer, it has been reported that ectopic expression of RUNX3 was observed in various cancers including breast cancer, glioma [8], [9]. Analysis of clinical tissue samples from peritoneal metastases arising from gastric cancers provides evidence that RUNX3 expression decreased significantly in the metastatic tissue, compared to normal gastric mucosa or primary main tumors.([10]) Importantly, the decrease in RUNX3 protein expression is significantly associated with poor survival of gastric cancer and melanoma patients [11], [12]. In our previous study, we demonstrated that RUNX3 can function as a tumor suppressor by regulating cell migration, invasion and angiogenesis in renal cell carcinoma [13]. These studies suggest a central role of RUNX3 in the tumorigenesis and progression. However, the function of RUNX3 in prostate cancer has not yet been well studied.

In this study, we examined the expression of RUNX3 in relation to clinicopathologic features using prostate cancer tissue microarray. We found that loss of RUNX3 expression directly correlated with prostate cancer TNM stage. In addition, restoration of RUNX3 expression led to repression of MMP-2 and induction of TIMP-2, which account for, at least in part, suppression of tumopr progression and metastasis. These results are consistent with the role of RUNX3 in regulating TIMP-2/MMP-2 in normal prostate cells. Furthermore, we demonstrated that decrease of VEGF secretion induced by RUNX3 reintroduction inhibited prostate cancer angiogenesis. Our clinical and mechanistic data indicated that RUNX3 may be a tumor suppressor involved in the progression of prostate cancer and targeting of RUNX3 pathway constituted a potential treatment modality for human prostate cancer.

## Materials and Methods

### Ethics Statement

This study of tissue microarray was performed under a protocol approved by the Institutional Review Boards of Affiliated Hospital of Xuzhou Medical College and all examinations were performed after obtaining written informed consents.

All animal experiments were performed using male nude mice (6–7 weeks old). The mice were purchased from the SLAC Laboratory Animal Ltd., Co. (Shanghai, China) and cared in accordance with the National Institutes of Health Guide for the Care and Use of Laboratory Animals. All animal experimental protocol was approved by Institutional Animal Care and Use Committee of Xuzhou Medical College (IACUC No. 1201).

### Patients and Samples

A prostate cancer TMA was purchased from Shanghai Xinchao Biotechnology (Shanghai, China). Tumors were staged according to the 2013 revised TNM system as follows [14]: 139 cases with stages I–II and 79 cases with stages III–IV. In addition, it includes 52 cases of tumor adjacent normal prostate tissue. The array dot diameter was 1.5 mm, and each dot represented a tissue spot from one individual specimen that was selected and pathologically confirmed.

### Immunohistochemistry

Immunohistochemistry staining were performed as described previously [13]. The primary rabbit anti-RUNX3 antibody (1∶200) (Medical and Biological Laboratories, Nagoya, Japan), anti-VIII (1∶100), anti-PAP (1∶100) (Santa Cruz, CA, USA) and the biotinylated anti-mouse IgG (1∶200) (Boster Biotech, Wuhan, China) were used. For the TMA staining evaluation, the immunoreactivity was assessed blindly by two independent observers using light microscopy (Olympus BX-51 light microscope), and the image was collected by Camedia Master C-3040 digital camera. The expression of RUNX3 was graded as positive when 10% of tumor cells showed immunopositivity. Biopsies with 10% tumor cells showing immunostaining were considered negative [15].

### Cell Lines and Transfection

Human prostate cancer cell lines PC3, DU145 and prostate cell RWPE-1 were purchased from the Shanghai Institute of Biochemistry and Cell Biology, Chinese Academy of Sciences (Shanghai, China). HUVECs were obtained from Nanjing Kaiji Biotech. DU145 cells were cultured in F12 medium supplemented with 10% fetal calf serum (FCS). RWPE-1 cells were maintained in K-SFM mediun with 10% FCS. PC3 cells and HUVECs were cultured in RPMI1640 medium supplemented with 10% FCS. Cells were in a 37°C humidified incubator with 95% air, 5% CO_2_. The pFlag-control and pFlag-RUNX3 expression plasmids were obtained from Dr Pei-Jung Lu (National Cheng-Kung University, Tainan, Taiwan).

For transient transfection, transfection of the pFlag-control and pFlag-RUNX3 plasmids into PC3 and DU145 cells were carried out using Lipofectamine 2000 transfection reagent (Invitrogen, Shanghai, China) following the manufacturer’s protocol. Transfection of the si-RUNX3, si-TIMP-2 and control siRNA into RWPE-1 cells were performed using siLentFect Lipid Reagent (Bio-Rad, Hercules, CA, USA). For stable transfection, the lentiviral expression vectors LV5-RUNX3 and LV5-Control were obtained from Shanghai Gene Pharma Company (China). The lentiviruses were mixed with polybrene (5 µg/ml) and added into DU145 cells for infection. The positive clones were selected in puromycin (5 µg/ml). The stable RUNX3 transfectants were isolated after 2 weeks during selection.

### Migration and Invasion Assay

Cell migration and invasion assay were determined by using a modified two chamber migration assay with a pore size of 8 µm. For migration assay, 1×10^6^ PC3 and DU145 cells were seeded in serum-free medium in the upper chamber. After 12 h incubation at 37°C, cells in the upper chamber were carefully removed with a cotton swab and the cells that had traversed the membrane were fixed in methanol, stained with hematoxylin and counted. For invasion assay, cells were seeded in serum-free medium in the upper chamber. After 24 h incubation at 37°C, noinvasive cells were gently removed from the top of the matrigel with a cotton-tipped swab. Invasive cells at the bottom of the matrigel were fixed in methanol and stained with leucocrystal violet. For quantification, cells were counted under a microscope in five fields (up, down, median, left, right. ×200).

### HUVEC Growth and Tube Formation Assay

Transfected PC3 and DU145 cells were cultured in 6-well plate with fresh complete medium for 24 h, the medium was collected and centrifuged to remove any cell debris before its use as a conditioned medium. For cell growth assay, 2×10^4^ HUVECs suspended in 100 µl conditioned medium and seeded at a density of 2×10^4^ in a 96-well culture plate and incubated for 24 h. Then, cell proliferation was detected using Cell counting kit-8 (CCK-8) purchased from Beyotime Institute of Biotechnology (Shanghai, China). For tube formation assay, 48-well plate was coated with Matrigel (BD Biosciences, NJ, USA) and kept in 37°C for 0.5 h. Then, 2×10^4^ HUVECs were suspended in 100 µl conditioned medium and applied to the pre-coated 48-well plate. After incubation at 37°C for another 24 h, the number of capillary-like tubes from three randomly chosen fields was counted and photographed under a Nikon inverted microscope (Nikon, Japan).

### ELISA for VEGF

PC3 and DU145 cells were plated in 6-well tissue culture plates at a density of 1×10^6^ cells per well. Then, cells were transfected with pFlag-control and pFlag-RUNX3 with serum starvation. The supernatants were collected 24 h after transfection. VEGF concentration was determined using Quantikine ELISA kits according to the manufacturer’s instructions (R&D Systems, MN, USA).

### Gelatin Zymography

Two million cells were seeded in 100-mm plate for 24 h. The proteins in the conditioned medium were harvested, placed on 10% SDS-PAGE containing 0.1% gelatin (Sigma, Shanghai, China). After electrophoresis, gel was incubated in Triton X-100 exchange buffer (20 mM Tris-HCl [pH 8.0], 150 mM NaCl, 5 mM CaCl and 2.5% Triton X-100) for 30 min followed by 10 min wash with incubation buffer (same buffer without Triton X-100) for 3 times. Gel was incubated in incubation buffer overnight at 37°C. After incubation, the gel was stained by 0.5% Coomassie Blue R-250 (Sigma, Shanghai, China) for 1 h, then de-stained in 30% methanol and 10% glacial acetic acid for 1 h. Gels were photographed and then quantitatedively measured by scanning densitometry.

### Quantitative Real-time PCR

mRNA was purified from cells using the RNeasy kit (Qiagen, Valencia, CA), and cDNA synthesis was performed with iScript Select cDNA Synthesis Kit (Biorad, Hercules, CA). Real-time PCR amplification of TIMP-2, MMP-2 and GAPDH was performed for 20 s at 95°C, followed by 50 cycles at 95°C for 3 s and annealing at 60°C for 30 s using an ABI PRISM 7500 Sequence Detection System (New York, USA). The results were normalized to those of the housekeeping gene GAPDH and are expressed as a ratio of the percentage of individual genes to the GAPDH control. The specific primer sequences for each gene are as follows: TIMP2 forward, TCTGGAAACGACATTTATGG; reverse GTTGGAGGCCTGCTTATGGG-3; TIMP2 forward, ACTGTTGGTGGGAACTCAGAAG; CAAGGTCAAT GTCAGGAGAGG and GAPDH forward, TGAA GGTCGGAGTCAACGGATT; reverse, CCTGGAAGATGGTGATGGGATT.

### Western Blot Analysis

Cell or tissue extracts were separated on a 10% SDS-polyacrylamide gel. The proteins were then transferred to nitrocellulose membrane and incubated overnight at 4°C with the following antibodies: mouse anti-RUNX3, rabbit anti-MMP-9, MMP-2, TIMP-1 and TIMP-2 (Cell Signaling Technology, MA, USA) and mouse anti-β-actin (Santa Cruz, CA, USA). Membranes were then washed and incubated with secondary antibody (goat anti-rabbit and goat anti-mouse IgG) for 2 h, stained by coloration fluid which contains 10 ml alkaline phosphatase buffer and developed using NBT/BCIP color substrate (Promega, Madison, USA).

### In vivo Experimental Metastasis Models

Two groups of 8 male nude mice were housed in SPF barrier facilities under a 12 h light/dark cycle. Mice were injected via tail vein with RUNX3 stably transfected DU145 cells (1×10^6^ cells per flank) in 100 µl of PBS. An insulin needle was used for the tail vein injections. Cell viability was determined by trypan blue exclusion and only single-cell suspensions >95% viable were used. Mice were euthanized at 8 weeks post-implantation, lungs were fixed in 10% formal dehyde, and hematoxylin and eosin (HE) stained for metastatic nodules.

### Subcutaneous in vivo Experiments

To evaluate tube formation, two groups of 8 male nude mice were injected with RUNX3 stably transfected DU145 cells (1×10^6^ cells per flank) in 100 µl PBS/Matrigel (50∶50) subcutaneously. The animals were monitored daily. The body weight of each mouse was recorded and tumor volume was determined by vernier caliper every day, following the formula of A×B^2^×0.52, where A is the longest diameter of tumor and B is the shortest diameter. Mice were euthanized at 8 weeks post-implantation, and tumors were surgically removed and fixed in 10% formal dehyde for histology. All experimental animal procedures were performed in compliance with the institutional ethical requirements and approved by the Committee of Xuzhou Medical College for the Use and Care of Animals.

### Statistical Analysis

Numerical data are expressed as means±S.D. Statistical differences between the means for the different groups were evaluated with Instat 5.0 (GraphPAD software, San Diego, CA) using one-way analysis of variance (ANOVA). For TMA, statistical analysis was performed with SPSS 11.5 software (SPSS). The association between RUNX3 staining and the clinicopathologic parameters of the prostate cancer patients, including age, tumor size, tumor grade and TNM stage, was evaluated by χ^2^ test. The significance of the in vivo data was determined using the two-tailed Mann-Whitney U test. Differences were considered statistically different at *P*<0.05.

## Results

### RUNX3 Expression is Reduced in Human Prostate Cancer

**Figure 1:**
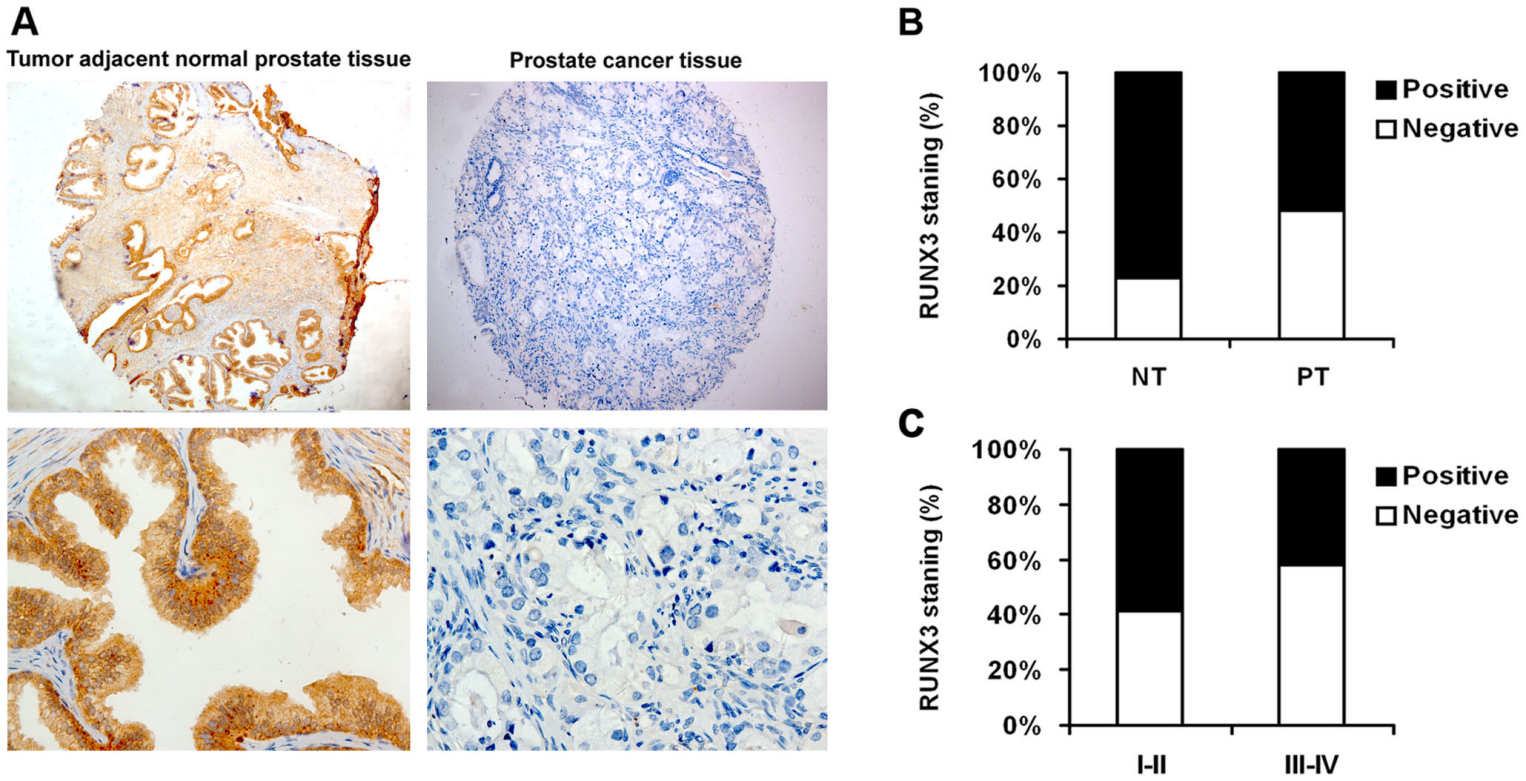
RUNX3 protein expression in tumor adjacent normal prostate tissues and prostate cancer tissues. A Representative immunohistochemical photographs were taken at different magnifications in tumor adjacent normal prostate tissue and prostate cancer tissues (Top panel ×100, bottom panel ×400). B Compared with that in the tumor adjacent normal prostate tissue, the overall expression level of RUNX3 in the prostate cancer tissues was significantly lower (*P*<0.01, χ^2^ test). C Decreased RUNX3 expression was correlated with TNM stage (*P*<0.01, χ^2^ test, comparing I-II versus III–IV).

We first determined whether RUNX3 expression is changed in human prostate cancer. Immunohistochemistry staining was performed in TMA slide containing tumor adjacent normal prostate tissues and prostate cancer tissues. The presentative pictures were presented in Fig. 1A. Our results showed that there was a significantly lower level of RUNX3 expression in the tumors than in the tumor adjacent normal prostate tissues (*P*<0.01, Fig. 1B). These suggested that RUNX3 was commonly expressed in normal human prostate tissue but decreased or absent in prostate cancer tissue.

### Loss of RUNX3 Expression in Prostate Cancer is Associated with Tumor Stage

In all 218 prostate cancer patients, the relationship between RUNX3 expression and pathologic and clinical features is shown in Table 1. The average (SD) age of patients was 58.7 years (median, 66.5 years; range, 37–87 years). Because TNM stage is an important prognostic marker for patients with prostate cancer, we detected the RUNX3 expression in 139 early-stage (I–II) and in 79 late-stage (III–IV) categories of prostate cancer tissues. We found RUNX3 staining was dramatically decreased in late stages when compared with early stage I–II (*P*<0.01, Fig. 1C). However, we did not find significant correlation between RUNX3 expression with other clinicopathologic variables, including age, tumor size and tumor grade.

**Table 1:** Patients characteristics and RUNX3 expression.

Variables	RUNX3 staining			
	Negative (%)	Positive (%)	Total	*P* *
**All cases Age**	113 (51.8)	105 (48.2)	218	
≤64 years	43 (45.7)	51 (54.3)	36	0.329
>64 years	65 (52.4)	59 (47.6)	39	
**Tumor size**				
≤2.5 cm	78 (51.3)	74 (48.7)	152	0.426
>2.5 cm	30 (45.5)	36 (54.5)	66	
**TNM stage**				
I	28 (44.4)	35 (55.6)	63	<0.01
II	33 (43.4)	43 (56.6)	76	
III	32 (59.3)	22 (40.7)	54	
IV	18 (72.0)	7 (28.0)	25	
**Grade**				
I	41 (52.6)	37 (47.4)	78	0.734
II	33 (47.8)	36 (52.2)	69	
III	23 (42.6)	31 (57.4)	54	
IV	11 (64.7)	6 (35.3)	17	

* *P* values are obtained from χ^2^ test.

### Restoration of RUNX3 Expression Inhibited Prostate Cancer Cells Migration and Invasion in vitro

**Figure 2:**
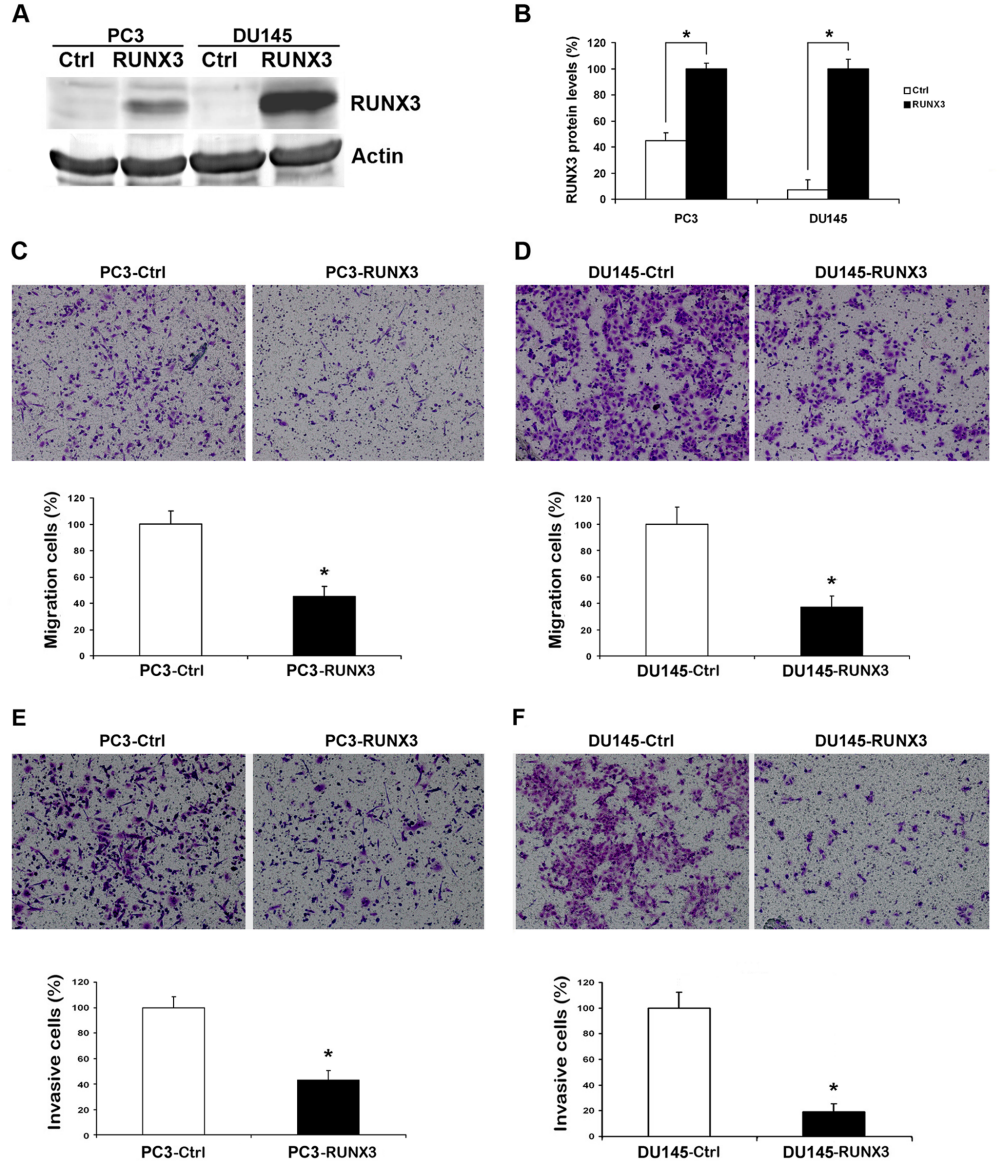
Reduction of RUNX3 on the abilities of metastasis in vitro. A and B Twenty-four hours after transfection, the expression of RUNX3 in PC3 and DU145 cells was evaluated by Western blot. β-actin was used as an internal control. C and D Cell migration assay. Representative fields of migration cells on the membrane (magnifications, ×200). Average migration cell number per field. E and F Matrigel cell invasion assays. Representative images show the cells that invaded through the Matrigel when transfected with RUNX3 plasmid or control. Representative histograph of invaded tumor cells is displayed and number of invaded tumor cells quantified. * indicates significant difference from the controls (*P<0.05, ANOVA).

Since RUNX3 expression is related to TNM stage with prostate cancer, RUNX3 may play important roles in one or more steps of prostate cancer metastasis. We first examine the effect of RUNX3 reintroduction on prostate cancer cells migration and invasion. We transiently transfected PC3 and DU145 cells with pFlag-control and pFlag-RUNX3 plasmids. Twenty-four hours after transfection, protein was significantly overexpressed in cancer cells (Fig. 2A and B). Transfected cells were subjected to cell migration assay and invasion assay. In cell migration assay, we found that RUNX3 restoration in PC3 and DU145 cells decreased the ability to migrate through Boyden chamber by 55% and 63%, respectively (Fig. 2C and D) (*P*<0.05). In cell invasion assay, RUNX3-transfected cells showed significantly lower invasive potency than the control, with invasive cells by 57% and 82% in PC3 and DU145 cells, respectively (Fig. 2E and F) (*P*<0.05). However, restoration of RUNX3 had no effect on the proliferation of prostate cancer cells (Data not shown).

**Figure 3:**
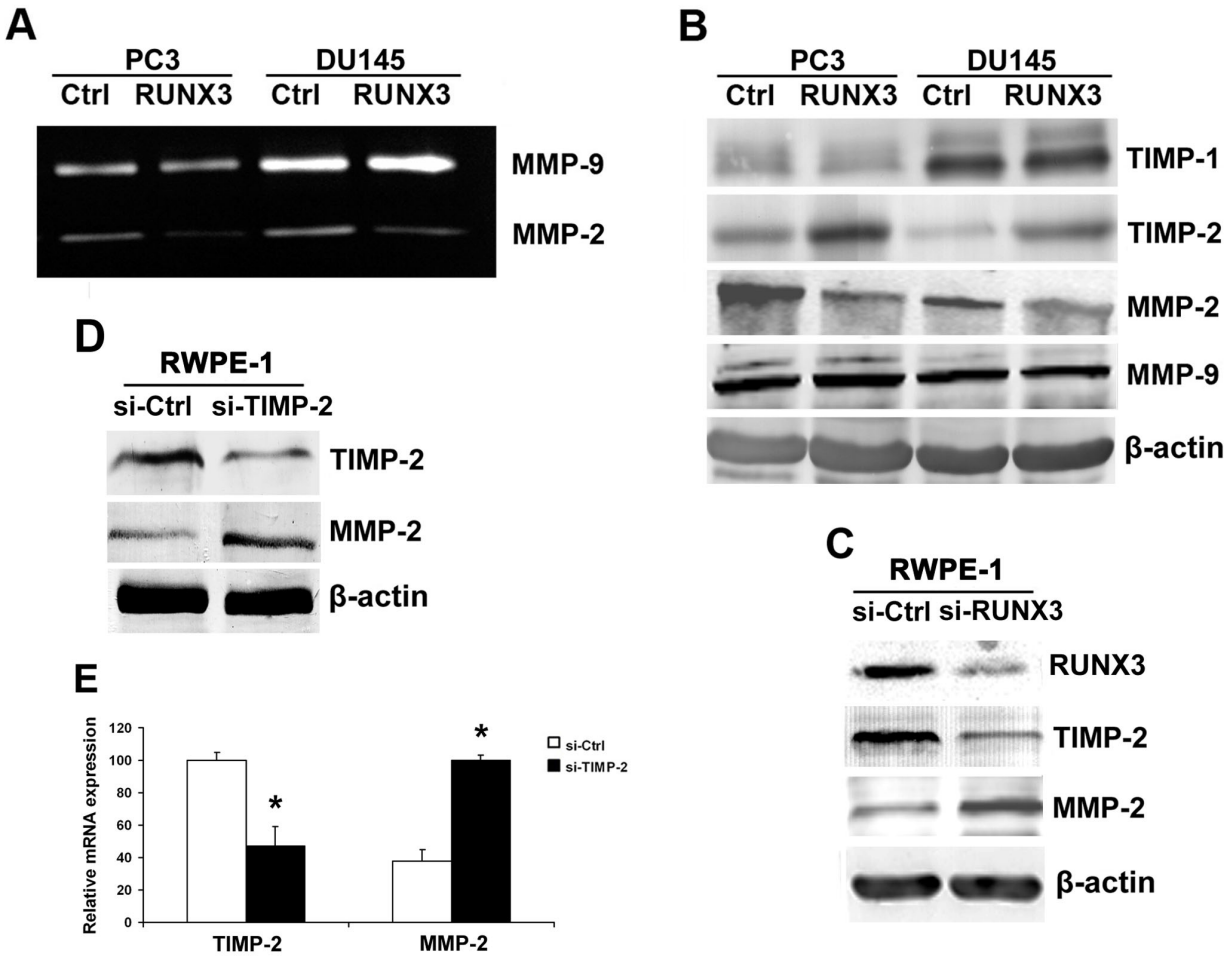
Target genes regulated by RUNX3. A The activity of MMP-2 and MMP-9 were evaluated by Gelatin zymography. B Western blot analysis of the relative protein levels of MMP-2, MMP-9, TIMP-1, TIMP-2 and β-actin in RUNX3 re-expression and control group for both PC3 and DU145 cell lines. C Western blot for the protein expression of MMP-2, TIMP-2 and β-actin in RUNX3 slicing and control group for RWPE-1 cell line. D Western blot analysis for MMP-2 and β-actin expression after knock down of TIMP-2. E Real time PCR for MMP-2 mRNA expression after knock down of TIMP-2. Data are shown as mean ± S.D. *P<0.05.

Invasive ability of cancer cells can be regulated by MMPs. To study the possible role of MMPs in RUNX3-induced inhibition of cell invasion, we performed gelatin zymography to measure the MMP-2 and MMP-9 activities. The MMP-2 enzyme activity was suppressed after forced expression of RUNX3 in PC3 and DU145 cells (Fig. 3A). Western blot was used to examine the TIMP-1, TIMP-2, MMP-2 and MMP-9 expressions in prostate cancer cells. Our results showed that the inhibition of MMP-2 protein level is due to the increased expression of TIMP-2 after RUNX3 overexpression in both cell lines (Fig. 3B). In order to further confirm the role of RUNX3 in regulating TIMP-2/MMP-2, normal prostate cell RWPE-1 was used in our study and our data showed that knock down of RUNX3 expression broke up the balance of TIMP-2/MMP-2 (Fig. 3C), while silence of TIMP-2 resulted in the inhibition of MMP-2 mRNA and protein expression in prostate cell (Fig. 3D and E). These results demonstrated that RUNX3 was involved in the regulation of TIMP-2/MMP-2.

### Restoration of RUNX3 Expression Reduced Angiogenesis in vitro

**Figure 4:**
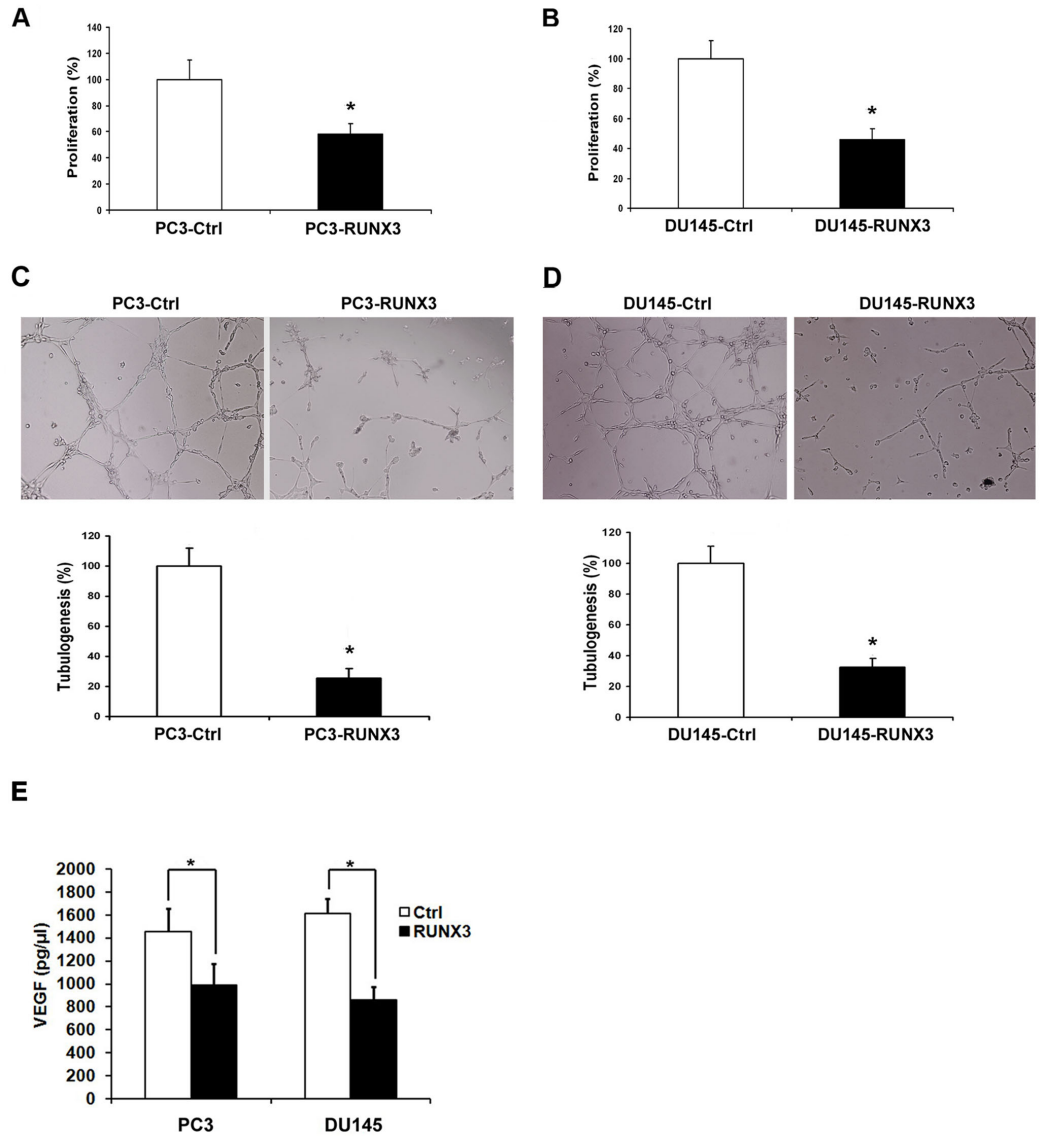
Inhibition of angiogenesis induced by RUNX3 expression. A and B CCK-8 cell proliferation assay was performed to detect the HUVECs proliferation. C and D Representative pictures were taken in situ for tube formation in the supernatant of PC3 and DU145 cells transduced with pFlag-control and pFlag-RUNX3. All experiments were carried out in triplicate. E ELISA for the secretion of VEGF in PC3 and DU145 cells transduced with pFlag-control and pFlag-RUNX3. Data are shown as mean ± S.D. *P<0.05.

To further determine the effect of restored RUNX3 expression on angiogenic potential of human prostate cancer cells, the angiogenic potentials of the supernatant of PC3 and DU145 cells transfected with pFlag-control or pFlag-RUNX3 were determined by endothelial cell proliferation assay and tube formation assay. In the cell proliferation assay, we found that conditioned medium from PC3 and DU145 cells transfected with pFlag-RUNX3 inhibited proliferation of endothelial cells compared with those of control cells (Fig. 4A and B). In the tube formation assay, the degree of tube formation was assessed as the percentage of cell surface area versus total surface area. As shown in Figure 4C and D, the average number of complete tubular structures formed by HUVECs was significantly decreased in conditioned medium from RUNX3 over-expressing PC3 and DU145 cells compared with vector controls (*P*<0.05).

VEGF is an important mitogen and survival factor for endothelial cells. In response to angiogenic stimulation, endothelial cells enter into an active proliferative state. To evaluate the mechanism of RUNX3 regulating angiogenesis, ELISA was performed to detect VEGF secreted into conditioned culture medium of prostate cancer cells. As shown in Fig. 4E, significant reduction in VEGF secretion was observed in conditioned medium from PC3 and DU145 cells transfected with pFlag-RUNX3 compared with control cells (*P*<0.05).

### Restoration of RUNX3 Expression Inhibited Metastasis in vivo

**Figure 5:**
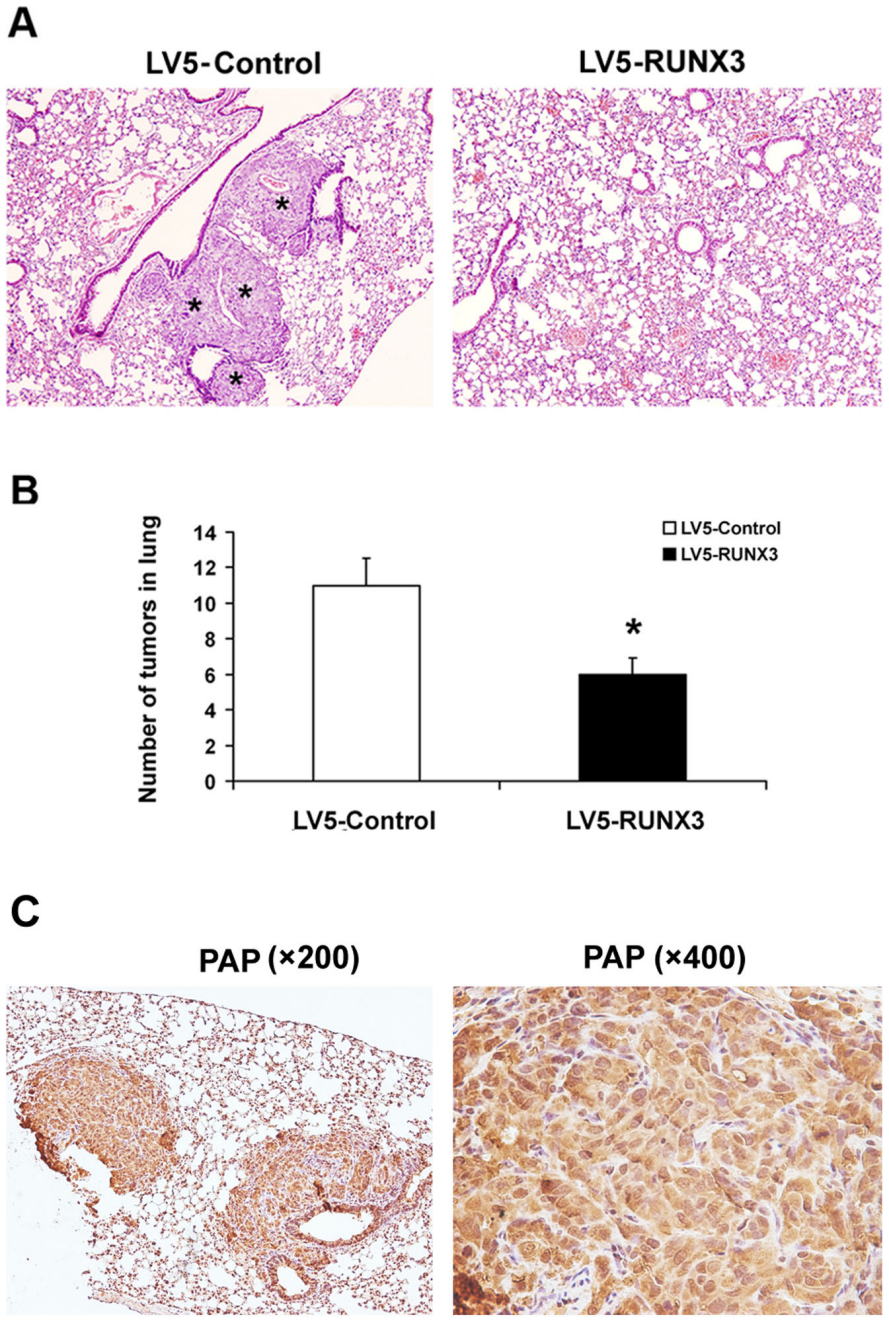
The up-regulation of RUNX3 repressed prostate cancer metastasis in vivo. A Mice were injected with were injected via tail vein with DU145 cells transfected with the indicated expression plasmids. Groups contained 8 mice. Eight weeks later, mice were sacrificed and the lung metastasis of DU145 cells was measured by macroscopic after autopsy. Histologic analysis of metastatic lesions in the ribs of nude mice was carried by HE staining. B Quantification of nodules in lung with altered RUNX3 levels. The numbers of nodules were blindly evaluated in 5 random fields per implant at 200 magnifications. Data are shown as mean ± S.D. *P<0.05. C Representative immunohistochemical photographs of PAP expression in lung tissue (magnifications, ×200 and ×400). *Tumor tissue.

Because RUNX3 expression decreased tumor cell migration and invasion in vitro, a tail vein metastatic assay was used to analyze the in vivo effects of RUNX3 expression on the metastatic potency of DU145 cells. Compared to control cells infected with empty virus vector, HE staining showed that tail vein injection of cells stably infected with LV5-RUNX3 into athymic nude mice led to significantly fewer number of nodules in the lung (Fig. 5A and B). To further confirm this distant metastasis, we performed immunohistochemistry to detect critical markers of prostate cancer. Prostate specific antigen (PSA) and prostatic acid phosphatase (PAP) are considered to be critical markers for diagnosis of prostate cancer [16]. However, PSA is not expressed in DU145 cells [17]. Thus, we used PAP in our study to investigate the source of tumor nodes in lung. As is shown in Fig. 5C, PAP positive in lung tissue indicated that the distant metastasis in the animal model was from the DU145 cells which were injected from tail vessel. These results showed the remarkable inhibitory effects of RUNX3 on pulmonary metastasis of prostate cancer cells.

### Restoration of RUNX3 Expression Suppressed Tumorigenesis in vivo

**Figure 6:**
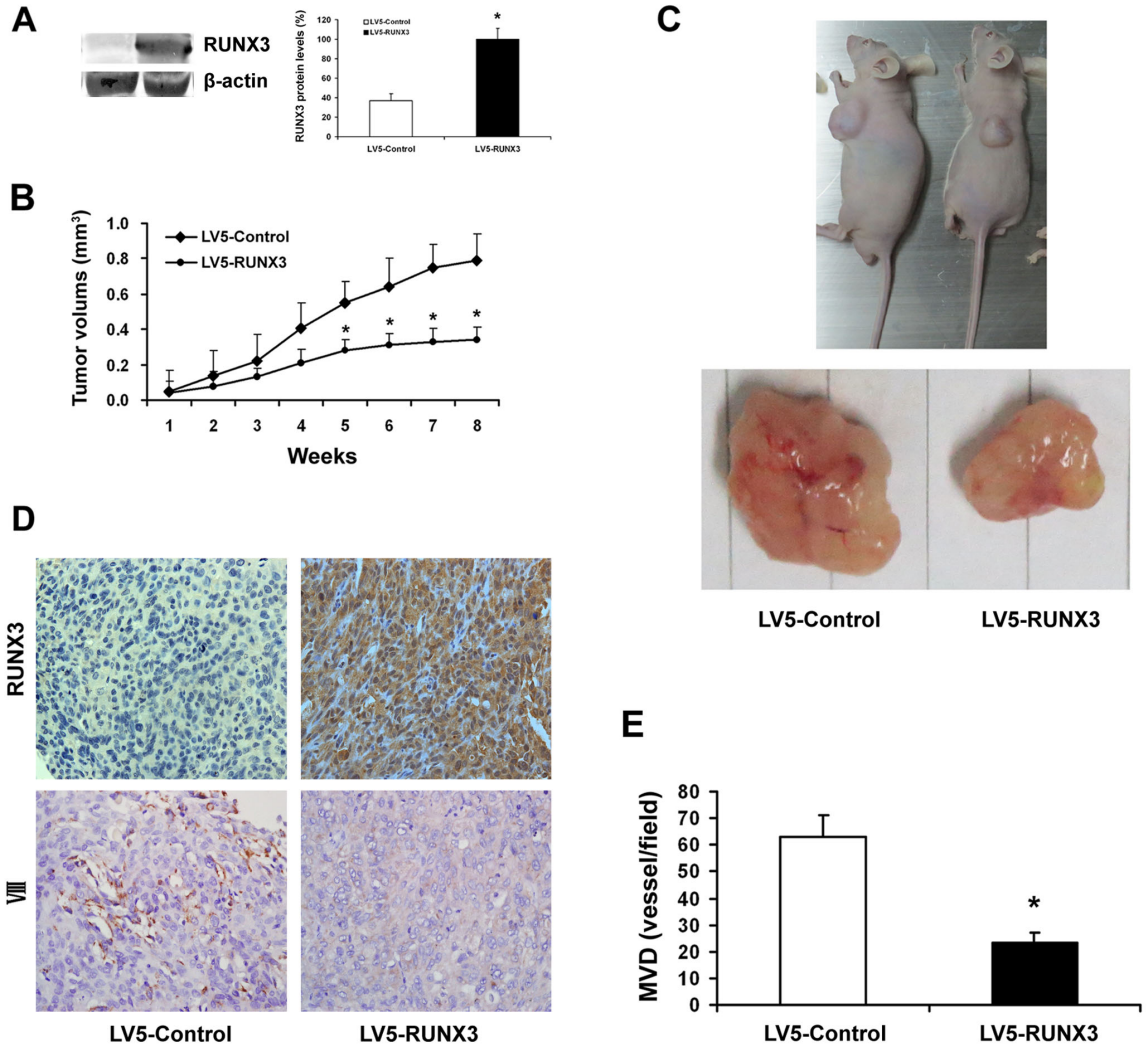
RUNX3 expression and angiogenesis in human prostate cancer growing in nude mice. A Nude mice were injected subcutaneously with DU145 cells stably expressing LV5-RUNX3 or LV5-Control. Western blot was used to examine RUNX3 protein level. B The tumors were monitored for total of eight weeks. Tumor diameter was measured with a caliper every week, and tumor volume was calculated following the formula of A×B^2^×0.52, where A is the longest diameter of tumor and B is the shortest diameter. The tumor volume was significantly greater at 8 week in mice given control lentiviruses compared with those given RUNX3 lentiviruses (*P<0.05). C Representative mice bearing tumors treated with control compared group with RUNX3 group. D The expression levels of RUNX3 and VIII were analyzed in tumor tissues by immunohistochemistry with representative images showed (magnifications, ×400). E Quantification of microvessel formation in tumors with altered RUNX3 levels. MVD was assessed via vessel counting. The numbers of stained microvessels were blindly evaluated in 5 random fields per implant at 200 × magnifications. * indicates significant difference from the controls (*P*<0.05).

To investigate the effect of RUNX3 treatment on tumor growth in vivo, we established a subcutaneous tumor in nude mice. Fig. 6A showed that RUNX3 protein was over-expressed after stably infected with LV5-RUNX3. Tumors in mice stably infected with LV5-RUNX3 had sustained a significant growth arrest compared with the controls (*P*<0.05). A representative photograph showing tumor growth in control and RUNX3 expressed mice (Fig. 6B).

To examine whether the suppression of tumor growth is associated with the effect of injected RUNX3 stably transfected cells, the tumors were dissected to examine RUNX3 expression by immunohistochemical analysis. As shown in the Fig. 6C, the RUNX3 protein was overexpressesed in RUNX3-injected xenograft compared with low RUNX3 expression in the control group. These results further strengthen the significant effect of RUNX3 suppression on prostate cancer growth.

### Restoration of RUNX3 Expression Reduced Angiogenesis in vivo

Since RUNX3 did not affect tumor cell proliferation in vitro, it is likely that RUNX3 suppressed tumorigenesis in vivo through blocking VEGF in endothelial cells (angiogenesis). We thus tested the effect of RUNX3 on angiogenesis. DU145 cells stably expressing RUNX3 or control vectors were mixed with Matrigel and injected into both flanks of the nude mice. The mice were sacrificed 56 days after implantation. The number of VIII-positive microvessels was much lower in the sections from xenografts of RUNX3-expressing DU145 cells (Fig. 6C and D), indicating that RUNX3 attenuated prostate cancer cell-inducing angiogenesis. The results suggested that the altered tumor growth and metastasis by restored RUNX3 expression was correlated with altered angiogenesis.

## Discussion

The role of RUNX3 in tumorigenesis has been studied extensively in recent years. Previous reports have shown that RUNX3 expression was reduced in numerous types of human cancers, such as breast cancer, colorectal cancer, renal cell carcinoma, melanoma [8], [11], [13], [18]. There has been evidence that RUNX3 methylation was especially frequent in different cohorts of prostate cancers but not in normal prostate mucosa [19], [20]. These observations suggest an important role for RUNX3 in human cancers, including prostate cancer. However, the function of RUNX3 in prostate cancer has not been examined. Better to understand the exact role of RUNX3 in prostate cancer development, we used TMA technology, in vitro cell model and in vivo animal model to investigate the role of RUNX3 in prostate cancer. Our clinical results showed that RUNX3 was lost in prostate cancer and correlated with TNM stage. Our in vitro studies revealed that RUNX3 in prostate cancer cells reduces cell migration, invasion and angiogenesis abilities, which was consistent with the function of RUNX3 in vivo.

Surgical resection is the most popularly used strategy in treatment with the primary prostate cancer [2]. However, for the patients with the advanced diseases, the surgical intervention shows less benifits [21]. Thus, it is essential to predict the risk of recurrence to minimize the adverse effects and maximize the therapeutic effect of treatment of prostate cancer. However, of the available prognostic factors for prostate cancer, the most important is the International Union Against Cancer TNM stage as determined by the depth of invasion, involvement of lymph nodes, and presence of distant metastasis [14]. In this study, we found that RUNX3 expression was lost in prostate cancer tissue. Moreover, RUNX3 protein was significantly reduced in late stage (Fig. 1). Our clinical evidence clearly supported the notion that altered expression of RUNX3 contributes to prostate cancer development and metastasis. It has been reported that reduced expression of RUNX3 was frequently caused by CpG island hypermethylation [19]. Moreover, point mutations of RUNX3 were observed in gastric and bladder cancers [22], [23]. However, the molecular basis of loss or decrease of RUNX3 expression in prostate cancer still remains to be determined.

Tumor cell migration and invasion are essential steps in the process of metastasis. In this study, we found that forced RUNX3 expression significantly inhibited prostate cancer cell migration and invasion abilities (Fig. 2). Furthermore, Overexpression of RUNX3 suppressed DU145 distant lung metastasis in nude mouse model, which suggested the tumor suppressor role of RUNX3 in prostate cancer metastasis (Fig. 5). MMPs are a family of zinc-dependent endopeptidase that are capable of degrading components of the basement membrane and extracellular matrix, allowing cancer cells to invade and migrate [24]. Many studies indicate that increased levels of MMP-2 or MMP-9 in the serum or tissue samples of prostate cancer patients are correlated with advanced stage [25], [26]. In addition, in vitro studies also show that the expression of MMP-2 and MMP-9 promote invasion and lymph node metastasis of prostate cancer cells [27]. We have previously demonstrated that RUNX3 decreased the activity of MMP-9 in renal cell carcinoma [13]. In the present study, we noticed that RUNX3 regulated prostate cancer cell metastasis through MMP-2 but not MMP-9. Chen *et al*. showed that MMP-9-inhibiting activity of RUNX3 due to the direct interaction of RUNX3 with the TIMP-1 promoter [28]. However, the mechanism of RUNX3 regulates MMP-2 expression and activity has remains to be unknown. MMP activity is controlled by specific, endogenous TIMPs [29]. TIMP2 is a main negative regulator of MMP2 enzyme activity and is involved in several tumor metastasis processes, including prostate cancer [30], [31]. There has been evidence that anti-invasive small molecule SR13179 block breast cancer cell metastasis via up-regulating TIMP2 expression [32]. In invasive human colorectal cells lines, treatment with the methyl donor S-adenosylmethionine (SAM) cause TIMP2 gene silence induced by promoter region CpG methylation, leading to inhibition of MMP-2 [33]. Transfecting human TIMP2 cDNA into human ameloblastoma cells caused xenograft growth inhibition in nude mice [34]. In the current study, we found that restoration of RUNX3 in prostate cancer cells simultaneously up-regulated TIMP-2 expression, which is the negative regulator of MMP-2 (Fig. 4A, B). Similarly, knock down of RUNX3 in non-cancerous prostate cells broke up the balance of TIMP-2/MMP-2, while silence of TIMP-2 resulted in inhibition of MMP-2 expression in prostate cell (Fig. 4C–E). These results suggested that the tumor suppressor ability of RUNX3 might be due to the elevated expression of TIMP-2, which subsequently inhibits the expression and activity of MMP-2.

Similar to other solid tumors, the growth and metastasis of prostate cancer depend on angiogenesis, which is the formation of new blood vessels from a pre-existing network of capillaries [35]. Angiogenesis is an attractive target in cancer therapy not only because it supplies oxygen and nutrients for the survival of tumor cells but also provides the route for metastatic spread of these cancer cells [36]. In this study, we found that RUNX3 has no effect on the proliferation of prostate cancer cells in vitro but inhibited tumor growth in animal model (Fig. 6A, B). We speculated that this is due to the tumor angiogenesis inhibition induced by RUNX3. Our in vitro and vivo results demonstrated that RUNX3 inhibited tumor growth by suppressing tumor angiogenesis (Fig. 6C, D). However, its molecular bases are unclear. Among several potential mechanisms, influences on the expression of various angiogenic molecules have been shown [37], [38], [39]. Obviously, the most essential angiogenic factors is VEGF, which exerts its mitogenic activity especially on endothelial cells [40]. VEGF has been identified as a key mediator of tumor angiogenesis involved in the development of tumor blood supply in the progression of human cancers [41]. VEGF expression is down-regulated by tumor suppressor genes p53, p75, and von Hippel-Lindau, which most likely occurs through their formation of complexes with Sp1 and inhibition of its binding to and transcriptional activation of the VEGF promoter [39], [42]. Peng *et al* also reported that VEGF expression is negatively regulated by RUNX3 via transcriptional repression in human gastric cancer [43]. Given the prominent role of VEGF in tumor angiogenesis, we detected the effect of RUNX3 overexpression on VEGF activity. Our data showed that VEGF secretion was decreased by restoration of RUNX3 in prostate cancer cells (Fig. 4C). Considering the facts that Sp1 activity is critical to VEGF expression and most oncogenes and tumor suppressor genes regulate VEGF expression via interaction with Sp1 [38], [42]. Sp1 signaling may also involved in the suppression of VEGF expression by RUNX3, but remains to be defined. Therefore, our results indicated that the angiogenic impairment was associated with the inhibition of VEGF expression induced by restoration of RUNX3.

In conclusion, this study provides evidence that decreased RUNX3 expression is significantly correlated with TNM stage of patients with prostate cancer. RUNX3 suppresses prostate cancer metastasis due to the imbalance between MMP-2 and TIMP-2. Furthermore, the inhibition of VEGF after RUNX3 restoration greatly contributed to tumor angiogenesis of prostate cancer cells in vitro and vivo. Thus, these findings identify RUNX3 as a promising novel therapeutic target for prostate cancer.
